# Hydrogel electrodes with conductive and substrate-adhesive layers for noninvasive long-term EEG acquisition

**DOI:** 10.1038/s41378-023-00524-0

**Published:** 2023-06-12

**Authors:** Hailing Xue, Dongyang Wang, Mingyan Jin, Hanbing Gao, Xuhui Wang, Long Xia, Dong’ang Li, Kai Sun, Huanan Wang, Xufeng Dong, Chi Zhang, Fengyu Cong, Jiaqi Lin

**Affiliations:** 1 Key State Laboratory of Fine Chemicals, School of Bioengineering, Dalian University of Technology, 116024 Dalian, China; 2 School of Biomedical Engineering, Faculty of Electronic Information and Electrical Engineering, Dalian University of Technology, 116024 Dalian, China; 3 Key Laboratory of Energy Materials and School of Materials Science and Engineering, Dalian University of Technology, 116024 Dalian, China

**Keywords:** Engineering, Bionanoelectronics

## Abstract

Noninvasive brain–computer interfaces (BCIs) show great potential in applications including sleep monitoring, fatigue alerts, neurofeedback training, etc. While noninvasive BCIs do not impose any procedural risk to users (as opposed to invasive BCIs), the acquisition of high-quality electroencephalograms (EEGs) in the long term has been challenging due to the limitations of current electrodes. Herein, we developed a semidry double-layer hydrogel electrode that not only records EEG signals at a resolution comparable to that of wet electrodes but is also able to withstand up to 12 h of continuous EEG acquisition. The electrode comprises dual hydrogel layers: a conductive layer that features high conductivity, low skin-contact impedance, and high robustness; and an adhesive layer that can bond to glass or plastic substrates to reduce motion artifacts in wearing conditions. Water retention in the hydrogel is stable, and the measured skin-contact impedance of the hydrogel electrode is comparable to that of wet electrodes (conductive paste) and drastically lower than that of dry electrodes (metal pin). Cytotoxicity and skin irritation tests show that the hydrogel electrode has excellent biocompatibility. Finally, the developed hydrogel electrode was evaluated in both N170 and P300 event-related potential (ERP) tests on human volunteers. The hydrogel electrode captured the expected ERP waveforms in both the N170 and P300 tests, showing similarities in the waveforms generated by wet electrodes. In contrast, dry electrodes fail to detect the triggered potential due to low signal quality. In addition, our hydrogel electrode can acquire EEG for up to 12 h and is ready for recycled use (7-day tests). Altogether, the results suggest that our semidry double-layer hydrogel electrodes are able to detect ERPs in the long term in an easy-to-use fashion, potentially opening up numerous applications in real-life scenarios for noninvasive BCI.

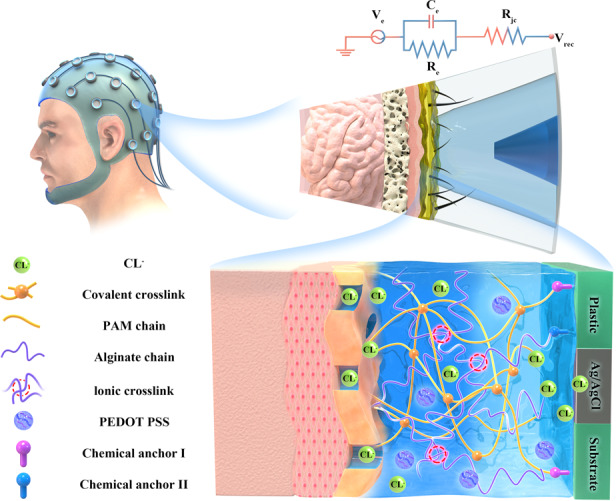

## Introduction

The brain–computer interface (BCI) is promising for establishing human‒machine interactions directly from the brain. By analyzing electrophysiological indices with the high spatial and temporal resolution, BCI can record brain activity in detail and convert the electroencephalogram (EEG) into machine instructions^1^. BCI has numerous potential applications, such as brain disease diagnostics, rehabilitation, disabled assistance, athlete training, health monitoring, and exoskeletons^2,3^. BCI can be categorized into noninvasive, semi-invasive, and invasive BCI, depending on the implantation method and the placement position of electrode sensors relative to the scalp^4^. Among these categories, noninvasive BCI can extract EEG without any procedural risk of infection or tissue injury, thus showing great potential for applications in daily scenarios.

The electrodes for noninvasive BCI to acquire EEG are usually divided into two types: dry electrodes and wet electrodes. Dry electrodes are composed of solid conducting materials, including metals, conductive fabrics, and conductive polymers, which can record physiological electrical signals by simply contacting the skin. However, dry electrodes have increased skin-contact impedance and reduced impedance stability, and thus, they exhibit higher susceptibility to motion artifacts. Other drawbacks of dry electrodes include increased signal noise and signal distortion, which are due to insufficient contact, ambient humidity, and sweat corrosion on the contact surface^5^. In addition, some dry electrodes, such as metal pins, can cause damage to the surface of the head^6,7^. Wet electrodes mostly comprise conductive paste and silver/silver chloride (Ag/AgCl), a material featuring high exchange current density, low polarization, and constant potential^8^. By using the conductive paste as the conducting medium, ion channels could be formed between the electrodes and ensure that the signal baseline remains smooth throughout the EEG recording. However, the setup procedure of wet electrodes with conductive paste is often complicated, time-consuming, and uncomfortable or even painful for users^9^. Additionally, for high-density electrode arrangements, the conductive paste may flow unexpectedly and cause short circuits between adjacent electrodes^10^. Moreover, wet electrodes cannot record EEG for more than a few hours due to the fast air drying of the conductive paste.

To address the abovementioned problems of noninvasive electrodes, researchers have proposed the concept of semidry electrodes. Semidry electrodes can utilize self-electrolytes to build ion channels at the electrode–skin interface, thus enabling reliable EEG signal collection comparable to wet electrodes and a more convenient setup similar to dry electrodes. Lee et al. developed a cellulose-based biosensor, which has high ion conductivity and is able to adjust secreted saline through self-regulation of the reservoir that holds NaCl saline solution embedded in the cellulose^11^. Duan et al. proposed a conductive nanocomposite network hydrogel fabricated by projection microstereolithography, which can probe EOG and EEG on bare skin without hair^12^. Paulo’s group proposed a novel semidry electrode prototype, which has the advantages of the low impedance, not requiring conductive paste, and not severely soiling or damaging the hair and skin of the head^13^. Pei’s group developed a microleakage electrode with an elastic tip that can continuously release electrolytes in a short period of time. However, the saline filling of this electrode may crystallize and aggregate after a few hours^14^. BrainCo Inc. applied semidry hydrogel electrodes to EEG products for attention monitoring, which can collect high-quality EEG signals on the forehead^15^. Compared with dry and wet electrodes, noninvasive semidry electrodes have lower contact impedance, a more convenient setup, and better-wearing comfort, thus providing a basis for next-generation BCI products^16^. However, semidry hydrogel electrodes still have several drawbacks that limit their application, including risks of easy water loss that elevates impedance, insufficient robustness and brittleness, and hydrogel securing issues in wearing condictions^1,17,18^. Currently, the acquisition of high-quality EEG in the long term is still challenging for noninvasive BCI.

In this manuscript, we propose a semidry double-layer hydrogel electrode that can acquire high-quality EEG signals for up to 10 h. The electrode has two layers. The first layer is composed of a conductive tough hydrogel that features low skin-contact impedance and high robustness capable of sustained usage. The second layer consists of an adhesive hydrogel that can bond to various substrate materials through chemical anchors. First, we described the formulation of the two hydrogel precursors and the fabrication of the double-layer electrode. We characterized the microscopic structure, mechanical strength, and bonding toughness of the hydrogel materials. We then characterized the electrical properties, including conductivity and skin-contact impedance, of the conductive hydrogel. The biocompatibility of the conductive hydrogel was also evaluated via cytotoxicity and skin irritation tests. Finally, we carried out EEG recording for event-related potential (ERP) detection using the developed electrodes on human volunteers. We compared the performance of our double-layer hydrogel electrodes with that of wet electrodes and dry electrodes in both N170 and P300 tests. The function and applicability of the proposed hydrogel electrode in real-life scenarios are then discussed.

## Materials and methods

### Chemicals

Sodium alginate (SA), N,N’-methylenebisacrylamide (MBAA), N,N,N,N-tetramethylethylenediamine (TEMED), and calcium sulfate dihydrate (CaSO_4_·H_2_O) were purchased from Adamas. Ammonium persulfate (APS), (3-aminopropyl)triethoxysilane (APTES), 3-(trimethoxysilyl)propyl methacrylate (TMSPMA), 1,6-hexamethylenediamine (HMDA) and benzophenone (BP) were purchased from Aladdin. Potassium chloride was purchased from Rhawn. Acrylamide (AM) was purchased from General Reagent. Glycerol and methanol were purchased from Damao. Poly(3,4-ethylenedioxythiophene)-poly(styrenesulfonate) (PEDOT:PSS) was purchased from Bidepharm. Isopropanol was purchased from Sangon Biotech. Ethanol was purchased from Chemical Reagent. All chemicals were used as received without further purification.

### Fabrication of conductive and adhesive hydrogels

To prepare the conductive hydrogel, 0.2 g SA, 1.2 g AM, and 0.7455 g KCl was dissolved in 10 ml deionized water. Then, 0.0066 g MBAA and 0.026 g CaSO_4_ were added as cross-linking agents after ultrasonic degassing. Both 0.004 g APS and 20 μL TEMED were added as initiators. Finally, 1.5 ml of PEDOT:PSS and 1.5 ml of glycerol were added and mixed quickly to synthesize a conductive hydrogel. The precursor solution was poured into a VeroClear mold, irradiated in an ultraviolet curing oven (UVHX300-400, 100% power) for 120 s, and then placed in an oven at 50 °C for 1 h to obtain a cured conductive hydrogel.

For the adhesive hydrogel, 0.2 g SA, 1.2 g AM, and 0.09 g KCl were dissolved in 10 ml deionized water and ultrasonically degassed. A total of 0.003 g MBAA and 0.026 g CaSO_4_ were added as cross-linking agents, 0.004 g APS was used as the initiator, 15 μL TEMED was used as the accelerator, and then 1 ml glycerol was quickly mixed to synthesize an adhesive hydrogel. The precursor solution was poured into a 3D-printed VeroClear mold, irradiated in a UV curing oven (UVHX300-400, 100% power) for 120 s, and then placed into an oven at 50 °C for 1 h to obtain a cured adhesive hydrogel.

### Chemical modification of substrates

Different substrate materials, such as glass, polypropylene (PP), polytetrafluoroethylene (PTFE), black rubber-like material (Rubber, TangoBlack FLX973), nylon (PA6), and polyvinyl chloride (PVC), were sequentially applied with isopropyl alcohol, ethanol, and deionized water for cleaning, followed by thorough drying. The cleaned substrate materials were treated with a plasma cleaner (TS-PL10, 100 W) for 5 min, immediately placed in the functional solution, and incubated at room temperature. After cleaning and thorough drying, the functionalized substrates were stored in low humidity conditions.

### Assembly of the hydrogel electrodes

Solidwork was used to design the matching supports and molds of the noninvasive conductive and substrate-adhesive double-layer hydrogel electrodes, and they were cast by 3D printing. After precleaning, plasma treatment, and the corresponding chemical grafting treatment, the printed supports of different materials were assembled with Ag/AgCl buttons and secured into molds. Noninvasive electrodes were prepared by injecting the prepolymer solution of the adhesive hydrogel and the conductive hydrogel into the appropriate position of the mold (the corresponding structure had been designed during the drawing) and then cured using both UV and thermal methods.

### Scanning electron microscopy

The microstructure of the conductive hydrogel was observed by a scanning electron microscope. The lyophilized sample was fixed on the sample stage with double-sided electrical tape and then sputtered twice with platinum. The morphology of the samples was then imaged with a scanning electron microscope (SU8200).

### Mechanical strength of the conductive hydrogel

Mechanical properties were measured using an MST, 50 N sensor. The compression test sample was cylindrical (10 mm in diameter, 8 mm in height), and the test rate was 5 mm/min. The 0–30% strain region was selected to calculate the compressive modulus.

### Bonding toughness of the adhesive hydrogel

The bonding toughness of the adhesive hydrogel was measured using the MST, 50 N sensor with a test rate of 15 mm/min. The test substrate materials were all cuboid: 75 mm × 25 mm × 2 mm, and the size of the adhesive hydrogel in the adhesive part was 15 mm × 25 mm × 4 mm. The TMSPMA-grafted glass substrate was used as the rigid substrate for the adhesive hydrogel. The shear bond strength value was calculated by dividing the original cross-sectional area by the maximum load at which the sample was peeled.

### Skin contact impedance

The four-electrode method was used to measure the contact resistance. The principle is shown in Fig. 3c. The implementation steps are as follows. The pretreated pigskins were removed from the −20 °C refrigerator and thawed for 10 min, and then the surface was washed with clean water to remove stains. The pigskin was cut into a rectangle with a length of 20 cm and a width of 5 cm, and it was flattened and fixed on the bottom plate of the measuring instrument. The four electrodes for measurement were arranged in a straight line, spaced 3.5 cm apart, fixed on the lower end of the measuring instrument slide plate, and located directly above the pigskin. Then, the circuit was connected according to the circuit diagram. The white slide was moved down to ensure that the electrodes were in close contact with the pigskin. The switch was turned to connect the *E*_b_-electrode to the circuit, adjusted to the appropriate voltage, and the data were recorded after the reading was stable. The same procedure was carried out with the *E*_a_-electrode connected to the circuit. The electrode–skin contact impedance was then calculated by the following formula:

$$Z_{{\rm {contact}}} =\frac{{V_{{\rm{bc}}}\times R}}{{V_{\rm{R}}}} -\frac{{V_{{\rm {bc}}}^\prime \times R}}{{V_{\rm {R}}^\prime }}$$

In the following experiment, a total of five different KCl concentrations (0.5 mol·L^−1^, 0.75 mol·L^−1^, 1.0 mol·L^−1^, 1.25 mol·L^−1^, 1.5 mol L^−1^) in the precursor of the conductive hydrogels were tested. The working voltage was set as 1 V, 2 V, 4 V, 6 V, 8 V, 10 V, 15 V, and 20 V. In addition, we also tested the contact resistance changes of wet electrodes and hydrogel electrodes continuously for 12 h at room temperature.

### Electrochemical impedance spectroscopy

Electrochemical impedance spectroscopy (EIS) was measured using a CHI660E workstation and tested in the frequency range of 0.1–10^6^ Hz. The test sample volume was 15 mm × 20 mm × 2 mm.

### Cell viability

Unless otherwise stated, mouse embryonic fibroblasts (3T3) were cultured in complete DMEM containing 10% fetal bovine serum and 1% penicillin/streptomycin at 37 °C in 5% CO_2_. The conductive hydrogel was prepared as described above. The gels were soaked in medium and removed at different time points to obtain different concentrations of conditioned medium, which were then analyzed for the effect of conditioned medium on cell growth. This experiment examines potential cumulative release or degradation products.

Effect of soaking duration on cells: Cylindrical gels (8 mm in diameter and 3 mm in thickness) were soaked in 8 ml of complete cell culture medium (DMEM, 10% fetal bovine serum, 1% penicillin/streptomycin) and placed at 37 °C in a 5% CO_2_ environment for cultivation. The gel was removed on days 1, 3, 5, 7, 10, and 14 to obtain conditioned medium of different concentrations. After the gel was removed, the medium was frozen, and after all time points were completed, the medium was thawed and used as a follow-up assayed cell culture medium.

The effect of monomer ratios on cells: Prepare circular gels (8 mm in diameter and 3 mm in thickness) with SA:AM ratios of 1:4, 1:5, 1:6, 1:7, and 1:8. The cells were soaked in 8 ml of complete cell culture medium (DMEM, 10% fetal bovine serum, 1% penicillin/streptomycin) and placed at 37 °C in a 5% CO_2_ environment for cultivation. The gel was removed on day 7 to obtain conditioned medium as cell culture medium for subsequent assays.

Cell proliferation assay: 3T3 cells were seeded in a 96-well plate at a density of 7500 cells/well, 100 μL of DMEM complete medium was added to each well, and the cells were cultured at 37 °C in a 5% CO_2_ environment for 20 h. The cell culture plate was removed, the cell culture medium was aspirated, the conditioned medium soaked in the gel was added, and the plate was incubated for 5 h at 37 °C in a 5% CO_2_ environment. In the dark, 10 μL of CCK-8 reagent was added to each well. After 1 h of dark incubation, the absorbance at 450 nm was measured with a microplate reader.

$$V_{{\rm {Cell}}} = \left[ {\frac{{{\rm {As}} - {\rm {Ab}}_1}}{{{\rm {Ac}} - {\rm {Ab}}_2}}} \right] \times 100{{{\mathrm{\% }}}}$$

As: Absorbance of experimental wells (including cells, medium, CCK-8 solution and drug solution); Ac: Absorbance of control wells (containing cells, medium, CCK-8 solution, without drugs); Ab_1_: Absorbance of blank well (containing culture medium, drug, CCK-8 solution, excluding cells); Ab_2_: Absorbance of blank well (containing culture medium, CCK-8 solution, excluding cells and drugs).

### Skin irritation test

To evaluate the biological response of the skin to the conductive hydrogel, we selected adult Japanese White Rabbits with an average body weight of 2 kg for the skin irritation test. The hair on the back of the rabbit was removed, and 4 square conductive hydrogel materials of 2 cm × 2 cm were applied externally on the bare skin for 1, 3, 5, and 7 days. After removing the accessories, we carefully observed whether the exposed skin was red, swollen, or seeped. After external application on the 7th day, the skin under the hydrogel was peeled off, fixed with paraformaldehyde, and stained with HE. Additionally, the unshaved back skin was chosen as a control.

### EEG acquisition

The EEG signals were collected from a 32-channel EEG signal acquisition system positioned in accordance with the international 10–20 lead system, with CPz as an online reference. The conductive-adhesive double-layer hydrogel electrodes were secured to the EEG cap through buttons. The subjects’ EEG signals were continuously collected using an eegoTM mylab amplifier (ANT Neuro, Enschede, Netherlands) with a sampling frequency of 1000 Hz. In addition, the real-time contact impedance of the electrodes is also recorded by the eegoTM software.

Three different electrodes, dry electrodes (waveguard touch, ANT Neuro), wet electrodes (Conductive Gel, Psytech)), and our hydrogel electrodes, were tested on human subjects for comparison of EEG signal acquisition. The setup of the test can be seen in Figs. 5a and 6a. The monitor in front of the subject performed a series of triggers, and an EEG cap (ANT Neuro) was mounted on the subject’s head and acquired an EEG signal during the tests. The tests were carried out in a soundproof room to reduce outside interference.

### N170 and P300 protocol

N170: In the case of ignoring the meaning of the word, the subjects responded to the key press within 2 s according to the color of the target word: red pressed the left button, blue pressed the right button. Before each target word appeared, there was a cross for 0.2 s and a blank space for 0.8–1.2 s, which was convenient for the subjects to focus their attention. Before the task started, there were six exercises to familiarize the subjects with the process. After reaching a 75% correct rate, the formal test began. Three healthy subjects were recruited for this experiment. Participants had guaranteed normal or corrected-to-normal vision and were not colorblind.

P300: This task required the subjects to visually search for the target element in the stimulus array when the stimulus pictures were presented. All stimuli were presented on the computer screen as white elements against a black background or white elements with some transparency. There were 16 elements in the stimulus array, including 14 perfect circles, 1 target item (vertical ellipse/horizontal ellipse), and 1 distractor item (horizontal ellipse/vertical ellipse). Each element in the stimulus array had a black origin inside, randomly located to the left or right of each element, as shown in Fig. 6d. The subjects were asked to search for the target item in the array within the time frame displayed by the stimulus array and used the left and right keys of the keyboard to feedback the position of the black dot in the target item. Each stimulus array had a white cross in the center of the screen for 500 ms, followed by a visual stimulus array presentation for 1000 ms, as shown in Fig. 6c. Twenty percent of stimulus arrays that did not contain target and distractor items appeared randomly during the experiment. Four healthy subjects were recruited for this experiment. Participants had guaranteed normal or corrected-to-normal vision and were not colorblind.

## Results and discussion

### Design and characterization of conductive and adhesive hydrogels

Conductive hydrogels usually comprise electrolytes or conductive polymer backbones. Electrolytes provide mobile ions inside the gel, whereas the polymer backbone employs electrons for signal conduction. The key to designing a conductive hydrogel for noninvasive BCI is increased robustness and high conductivity. To that end, we chose a hydrogel system with a double crosslinking network (sodium alginate, SA and polyacrylamide, PAM) for increased robustness. On the other hand, we adjusted the ratio of electrolyte content (water and potassium chloride, KCl) in the hydrogel precursor for enhanced ion mobility to achieve high conductivity. Glycerol was added to the hydrogel precursor for increased moisturization and the prevention of water loss. The hydrogel was synthesized via in situ polymerization. We also adjusted the ratio of SA and AM monomers to further increase the robustness of the gel. Finally, we added a conductive polymer, poly(3,4-ethylenedioxythiophene) (PEDOT) and poly(styrene sulfonate) (PSS), to the gel precursor to facilitate electron conduction inside the gel in addition to mobile ions^19–21^.

**Fig. 1:**
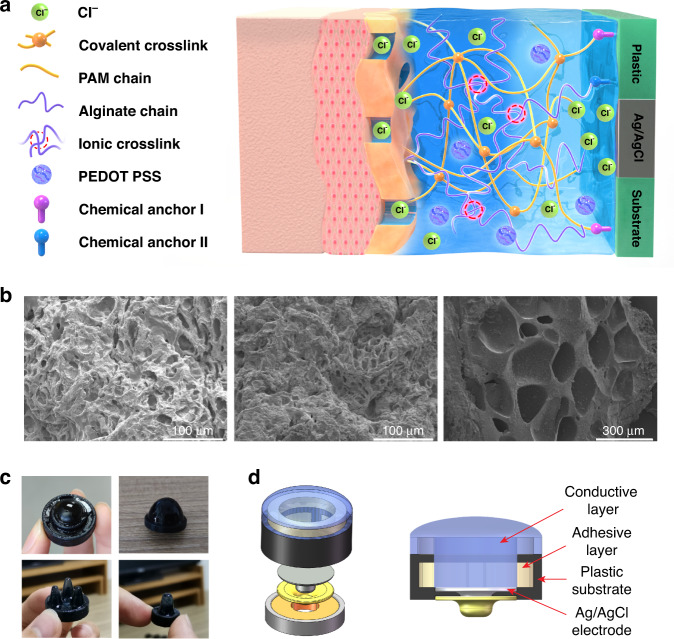
Design strategy for the double-layer hydrogel electrodes. **a** The structure of the conductive-adhesive double-layer hydrogel. The conductive hydrogel acquires bioelectric signals on the gel-tissue interface via mobile chloride ions through micro channels on the epidermis. **b** SEM image of the conductive hydrogel. **c** Casted hydrogel electrodes with different ergonomic designs to maximize contact with the scalp. **d** Assembly of the hydrogel electrode with a conductive layer, an adhesive layer, supports, and Ag/AgCl

In addition to the conductive hydrogel, we also formulated an adhesive layer of hydrogel in the electrode. The adhesive layer has similar constituents to that of the conductive layer but with less water, no electrolyte and PEDOT:PSS, providing it with even higher toughness. After UV curing and thermal curing, both the conductive hydrogel and adhesive hydrogel were obtained in a combined manner. As shown in Fig. 1a, both the conductive hydrogel and adhesive hydrogel used acrylamide (AM) and SA as monomers to form a double interpenetrating polymer network featuring covalent crosslinks and ionic crosslinks. The two types of hydrogels were seamlessly combined by the double network via polymerization. The adhesive hydrogel is bound to the substrate through chemical anchors by either PAM or alginate chains. The conductive hydrogel is in direct contact with the epidermis, detecting EEG signals from the dermis and the underlying tissue via mobile chloride ions through microchannels formed in the epidermis. On the other hand, the conductive hydrogel is in contact with Ag/AgCl electrodes, and the electric signal is transmitted from the epidermis to Ag/AgCl via chloride ions moving in the hydrogel.

The surface and internal structure of the conductive hydrogel were further investigated by scanning electron microscopy. As shown in Fig. 1b, a rich porous texture is found on the surface of the conductive hydrogel (left and middle images). On the other hand, larger pores up to hundreds of microns are found on the cross-section of the conductive hydrogel, indicating that the gel has relatively higher water content.

Through ergonomic design, we cast several hydrogel electrodes with different shapes to maximize the contact of the gel surface to the hairy scalp (Fig. 1c). In all of these different designs, the assembly of the double-layer hydrogels on the substrate is the same. In the assembly, the lower part of the conductive hydrogel is surrounded by the adhesive hydrogel, forming a ring shape, and is firmly attached to the inner surface of the plastic substrate (Fig. 1d). The bottom part of the conductive hydrogel is in contact with Ag/AgCl, which is further attached to a 4-mm snap. Through this assembly, the conductive hydrogel can stably acquire EEG signals in an easy-to-go fashion using standard EEG headsets.

**Fig. 2:**
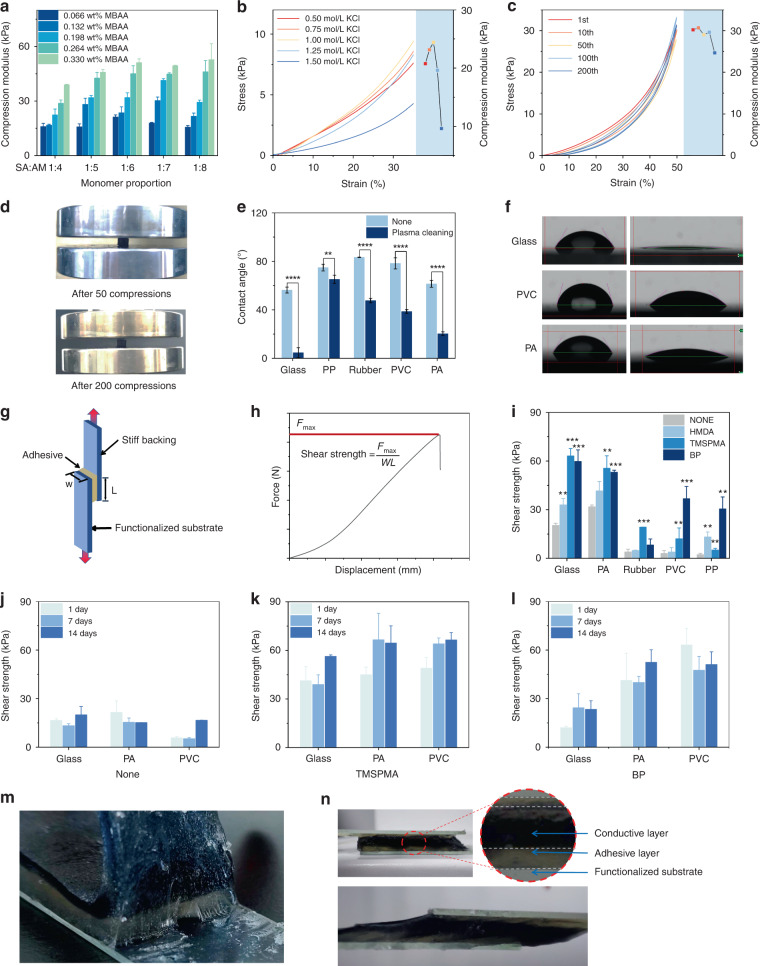
Mechanical and bonding properties of the conductive and adhesive hydrogel. **a** Compressive modulus of conductive hydrogels with different MBAA contents and different SA: AM ratios. **b** Stress‒strain curves of conductive hydrogels with different KCl concentrations. **c** Stress‒strain curves of the conductive hydrogel with 200 cycles of compression. **d** Images of the conductive hydrogel after 50 cycles and 200 cycles of compression. **e** Contact angle of solid substrates before and after plasma cleaning. **f** Contact angle images of glass, PVC, and PA before (left) and after plasma cleaning (right). **g** Schematic diagram of lap-shear tests. **h** Equations of shear strength calculation. **i** Tested shear strength of the adhesive hydrogel on glass, PA, Rubber, PVA, and PP. **j** Shear strength of the adhesive hydrogel on three solid substrates without chemical treatment at 1, 7, and 14 days. **k** Shear strength of the adhesive hydrogel on three solid substrates treated by TMSPMA at 1, 7, and 14 days. **l** Shear strength of the adhesive hydrogel on solid substrates treated with BP at 1, 7, and 14 days. **m** Adhesion-conductive bilayer hydrogels bonded to TMSPMA-treated glass. **n** The interface between the adhesive and the conductive layers

To explore the effects of different monomer ratios on the mechanical properties of the conductive hydrogels, we performed a series of compression tests. The stress‒strain curves of the conductive hydrogels with different MBAA and monomer contents are shown in Fig. 2a. With sufficient MBAA dosage, the mechanical strength of the conductive hydrogel increases monotonically with an increase in the SA-to-AM ratio. This can contribute to the increase in the cross-linked PAM network, which provided rigid support for the double-network conductive hydrogel and consequently increased toughness^22,23^. When the amount of MBAA was insufficient, the compressive modulus of the conductive hydrogels peaked at a 1:6 SA-to-AM ratio and decreased with a further increase in the ratio. This is probably because under an insufficient MBAA dosage, the cross-linking degree of PAM reached a plateau at a 1:6 ratio, and further increasing the amount of AM did not contribute to the PAM network. Considering the long-term wearing comfort of the hydrogel electrode, we chose a ratio with a moderate compressive modulus (~20 kPa), which is closer to that of human tissue^24^.

We then measured the compressive modulus of the conductive hydrogel with different KCl concentrations. Under a 1:6 SA-to-AM ratio, the compressive modulus showed a trend of initially increasing and then plummeting with increasing KCl concentration (Fig. 2b). Based on the Hofmeister effect, the solubility of hydrophilic polymers in water is affected by salt ions. When an appropriate concentration of KCl was introduced into the conductive hydrogel, the three-dimensional network structure was denser due to the physical entanglement of KCl. On the other hand, a large KCl concentration affected the bond between water and polymer. The dissolution of K^+^ and Cl^-^ made the network cavity in the conductive hydrogel larger, and consequently, the degree of cross-linking decreased. Therefore, the compressive modulus decreases substantially with a further increase in the KCl concentration^25–27^.

To test the anti-fatigue and rapid self-recovery ability of the conductive hydrogel when it is continuously subjected to compressive stress, we conducted 200 cyclic compression tests on the conductive hydrogel (Fig. 2c, d). The curves of the selected cycles (1st, 10th, 50th, 100th, and 200th) almost completely overlap, and there is no obvious hysteresis. The corresponding compressive modulus only shows a slight drop. This demonstrates that the network structure of the conductive hydrogel was not broken during the continuous 200-cyclic compression process and had good self-recovery and fatigue resistance. This is due to the large number of reversible molecular interactions in these conducting hydrogel networks, such as the ionic cross-linking formed by alginate and Ca^2+^. In addition, hydrogen bonds and electrostatic interactions in the gel system also promoted physical cross-linking, which can dissipate external energy^28,29^.

Next, we investigated the bonding between the adhesive gel and several different solid substrates, including glass, rubber, PA, PVC, and PP. After precleaning and oxygen plasma treatment, the surface of these substrate materials became rougher, and hydroxyl groups were obtained on the surface, which easily reacted with functional solutions and thus better adhered to the hydrogel^30^ (Fig. 2e, f). To test the bonding toughness of the adhesive hydrogel to the chemically treated material substrate, we performed several lap-shear tests. The setup of the lap shear test is shown in Fig. 2g, h.

After different chemical treatments of solid substrates (1,6-hexamethylenediamine (HMDA), TMSPMA, 3-(trimethoxysilyl)propyl methacrylate (TMSPMA) and benzophenone (BP)), the adhesive properties of the adhering hydrogels were significantly improved compared to untreated substrates. (Fig. 2i). TMSPMA easily formed hydrogen bonds with hydroxyl groups on the surface of the material in acidic aqueous solutions, grafted functional silanes to the surface of solid substrates, and then copolymerized with acrylate groups in the adhesive hydrogel to form chemical anchoring^31^. BP is often used as a photoinitiator and can be physiosorbed to the substrate surface during incubation at room temperature. It can generate free radicals during the UV curing of hydrogels, thereby forming covalent interconnections between the adhering hydrogels and solid substrates^32–34^. HDMA can be used as a bridging polymer, which can physically entangle the surface of solid substrates in aqueous solution and covalently couple the carboxylic acid groups in the adhesive hydrogel with the primary amine groups on the functionalized substrates^35^. Among these treatments, TMSPMA had the most significant impact on glass, PA, and rubber. BP shows the most significant impact on PVC and PP. HDMA shows a limited effect on all five substrate materials. For the sustainable use of hydrogel electrodes, the long-term stability of the bonding of the adhesive hydrogel to the solid substrate is crucial. We selected glass, PA, and PVC, which show good shear strength with the hydrogel, used TMSPMA and BP as chemical treatments, and measured the shear strength between the gel and the substrate on the 1st, 7th, and 14th days after curing. The results are shown in Fig. 2j–l. The shear strength of the adhesive hydrogel did not decrease over time, indicating that the adhesion of the hydrogel to the solid substrate has long-term stability. For the adhesion between the adhesive hydrogel and the conductive hydrogel, due to the similar components of the two hydrogels, they had no obvious delamination interface after curing, and they were completely integrated, as shown in Fig. 2n.

### Water retention, impedance, and biocompatibility

**Fig. 3:**
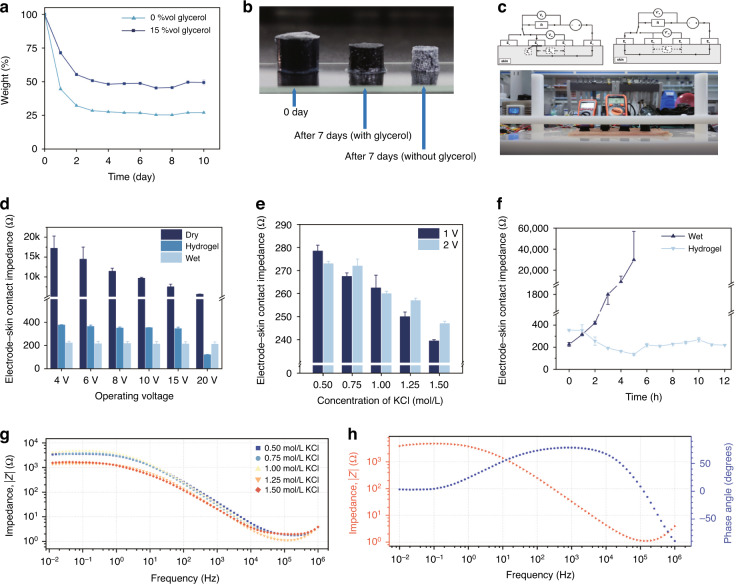
Water retention ability and electrode-skin contact impedance of the hydrogel. **a** Water retention of the conductive hydrogel with and without glycerol over 10 days. **b** The appearance change of the conductive hydrogel after 7 days of exposure to air. **c** Setup of the measurement of the electrode–skin contact impedance using model animal skin. **d** Skin contact impedance of dry electrode, double-layer hydrogel electrode, and wet electrode under different operating voltages. **e** Skin contact impedance of conductive hydrogels with different KCl concentrations. **f** Skin contact impedance of the wet electrode and double-layer hydrogel electrode for 12 h of continuous monitoring. **g** Electrochemical impedance spectroscopy of the conductive hydrogel with different KCl contents across a wide range of frequencies. **h** Electrical impedance and phase angle of the conductive hydrogels across different frequencies

Water retention ability is critical for semidry hydrogel electrodes since their conductivity is directly correlated with the amount of water content inside. In some studies, hydrogels can completely dry if exposed to air for a long period of time^20,36^. Here, we assessed the water retention ability of the conductive hydrogel by measuring its weight. Conductive hydrogels with and without glycerol were both tested. The weight of the conductive hydrogel with glycerol decreased after fresh curing, became stable after 3 days of exposure to air, and was able to retain more than half of its water content. In contrast, the hydrogel without glycerol shrank to ~25% of its mass after 3 days of exposure to air (Fig. 3a). Figure 3b shows that the hydrogel with glycerol maintained its normal appearance after 7 days, whereas the hydrogel without glycerol was dehydrated and dried.

Electrode–skin contact impedance is one of the most important characteristics of noninvasive electrodes. It is generally considered that the contact impedance of wet electrodes should be <5 kΩ, whereas the contact impedance of dry electrodes is in the range of ~100 kΩ. Here, using model animal skin, we measured the skin contact impedance of wet (paste-based), dry (solid-pin), and our double-layer hydrogel electrodes. The setup of the impedance measurement is shown in Fig. 3c. In the range of the voltage tested (<20 V), the skin contact impedance of the wet and hydrogel electrodes is <0.4 kΩ, whereas the impedance of the dry electrodes is up to ~15 kΩ (Fig. 3d). In addition, the impedance changes under different test voltages for the hydrogel electrode were quite small, indicating that the semidry hydrogel electrode can stably collect bioelectric signals. Next, we investigated the effect of KCl concentration on the contact impedance of the hydrogel electrode. With increasing KCl concentration in the conductive hydrogel, the electrode–skin contact impedance showed a downward trend (Fig. 3e). This can be attributed to the high concentration of KCl electrolyte in the hydrogel electrode, which increased the ion transfer efficiency on the skin–electrode interface and resulted in a decrease in contact impedance. The long-term impedance stability of wet and hydrogel electrodes was also tested. The hydrogel electrode can remain under working conditions (<0.4 kΩ) for up to 12 h, whereas the impedance of the wet electrode rapidly increased to 10 kΩ in 4 h and became infinitely large after 5 h due to drying out (Fig. 3f).

The electrochemical impedance spectroscopy of the hydrogel electrode was also tested across a wide range of frequencies using an electrochemical workstation^37^. Generally, the impedance drops with increasing testing frequency (Fig. 3g, h). The conductive hydrogel exhibited low impedance (<0.25 kΩ) at physiologically relevant frequencies (10^2^–10^5^ Hz). At EEG-related frequencies (0.5–50 Hz), its maximum impedance value was <5 kΩ. At low frequencies, a high KCl concentration can effectively reduce the impedance of the conductive hydrogel.

**Fig. 4:**
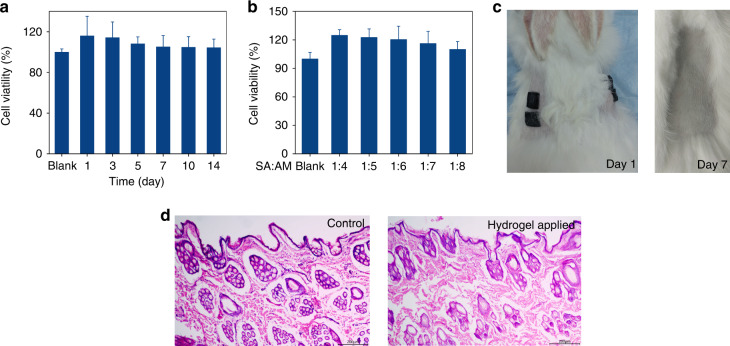
Biocompatibility test of the conductive hydrogel. **a** Cell viability results of conducting hydrogel in DMEM complete medium for 14 days (*n* = 6). **b** Cell viability results of the conductive hydrogel with different SA:AM ratios placed in DMEM complete medium for 7 days (*n* = 4). **c** Skin irritation test of the conductive hydrogel. **d** Images of hematoxylin and eosin (H&E)-stained tissues of the skin without conductive hydrogel (left) and after 7 days of conductive hydrogel application (right)

Since the hydrogel electrode should be in contact with the human epidermis under working conditions, the biocompatibility of the hydrogel material is crucial in long-term EEG recording. Therefore, we carried out cell viability and skin irritation tests on the conductive hydrogel. Figure 4a shows the cell viability of the conductive hydrogels soaked for different periods of time. For all soaking durations tested, the cell viability was more than 100% compared to the control, indicating that the conductive hydrogel is not cytotoxic. Figure 4b shows the cell viability of the conductive hydrogels with different monomer ratios in DMEM complete medium for 7 days. As the SA:AM ratio increased, the cell viability was slightly reduced. However, overall, no significant difference in viability was observed.

Next, we performed skin irritation experiments on the conductive hydrogel to further explore its biocompatibility. Using a Japanese White Rabbit as a testing animal, we removed the hair on its back and applied the gel to the bare skin. The skin surface where the conductive hydrogel was applied did not have any redness, swelling, or exudation (Fig. 4c). Further examination of the contact tissue was conducted. Figure 4d shows a representative image of hematoxylin and eosin (H&E)-stained tissue at the application site, and no inflammatory reaction was found (right). These results confirm that the conductive hydrogel material is highly biocompatible.

### EEG signal acquisition for ERPs

**Fig. 5:**
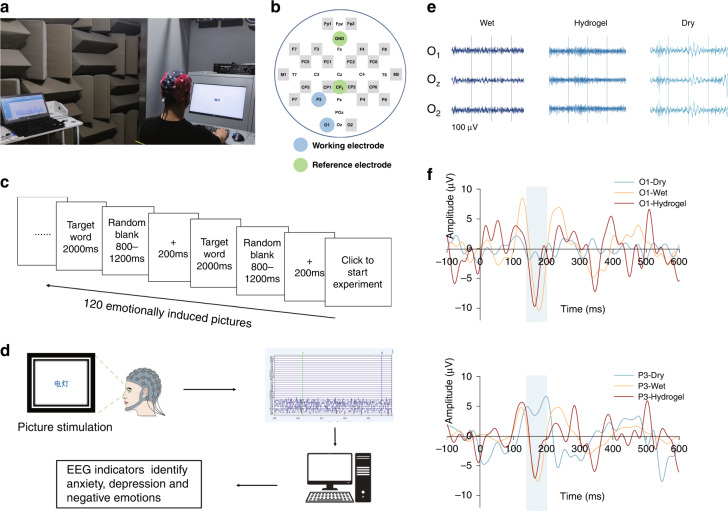
N170 test using wet, dry, and hydrogel electrodes by an EEG cap. **a** A photograph of a subject being tested during EEG monitoring to evoke an N170 response. **b** Location of two working electrodes and two reference electrodes for EEG measurements in the EEG cap. **c** Schematic diagram of the picture stimulation flow. **d** The experimental function diagram of the N170 test. **e** EEG raw signals of wet, conductive hydrogel, and dry electrodes in Experiment 1. **f** N170 waves at O1 and P3 for dry, wet, and double-layer hydrogel electrodes (*n* = 3)

**Fig. 6:**
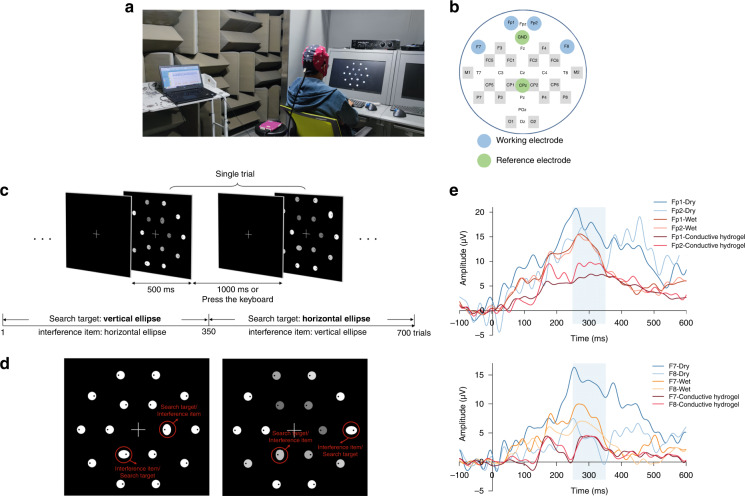
P300 test using dry, wet, and hydrogel electrodes by an EEG cap. **a** A photograph of a subject being tested during EEG monitoring to evoke a P300 response. **b** Location of four working electrodes and two reference electrodes for EEG measurements. **c** Schematic diagram of the picture stimulation flow of Experiment 2. **d** Visual stimulus pictures with target items. **e** P300 waves at O1 and P3 for dry, wet, and double-layer hydrogel electrodes (*n* = 4)

Event-related potentials (EPRs) are effectively used for evaluating brain functions by measuring EEG. In terms of signal acquisition, ERP provides a practical way to identify the efficacy of noninvasive BCI electrodes^38^. Here, we conducted both the N170 BCI test and P300 BCI test with wet, dry, and double-layer hydrogel electrodes on human volunteers in a soundproof environment (Figs. 5a and 6a). In the N170 BCI test, two electrode positions, O1 and P3, were selected for analysis (Fig. 5b). The protocol of the simulation flow and the experimental diagram are given in Fig. 5c, d. The acquired raw EEG signals for three different electrodes are shown in Fig. 5e. The hydrogel electrode and wet electrode share a similar raw signal form, while the dry electrode shows a higher noise level. For the triggered potential response, both wet and hydrogel electrodes show the expected negative peaks at approximately 170 ms, indicating successful detection of the ERPs (*n* = 3, averaged). Furthermore, the EEG waveforms collected by hydrogel electrodes were very close to those of the wet electrode, and the time difference between the peaks of the two was less than 13 ms. On the other hand, the waveform of the dry electrode is quite different from that of the wet and hydrogel electrodes, and there is no obvious negative peak within the time window of N170 (Fig. 5f).

In the P300 test, four electrode positions, F7, F8, FP1, and FP2, were selected for analysis^39–41^ (Fig. 6b). The stimulation flow and the visual stimulus picture are shown in Fig. 6c, d. The experiment is conducted on four subjects, and the amplitude waveforms are averaged (Fig. 6e). Both the double-layer hydrogel electrode and the wet electrode showed a triggered response approximately 300 ms after the start of stimulation, although there was an amplitude difference between the two. The amplitude of the P300 component was initially small, but there was a very obvious increase in the amplitude at 300 ms. In contrast, the waveform of the EEG signal collected by the dry electrode fluctuated greatly, and one can barely identify the P300 component, indicating that the dry electrode is not potent for the detection of ERPs.

**Fig. 7:**
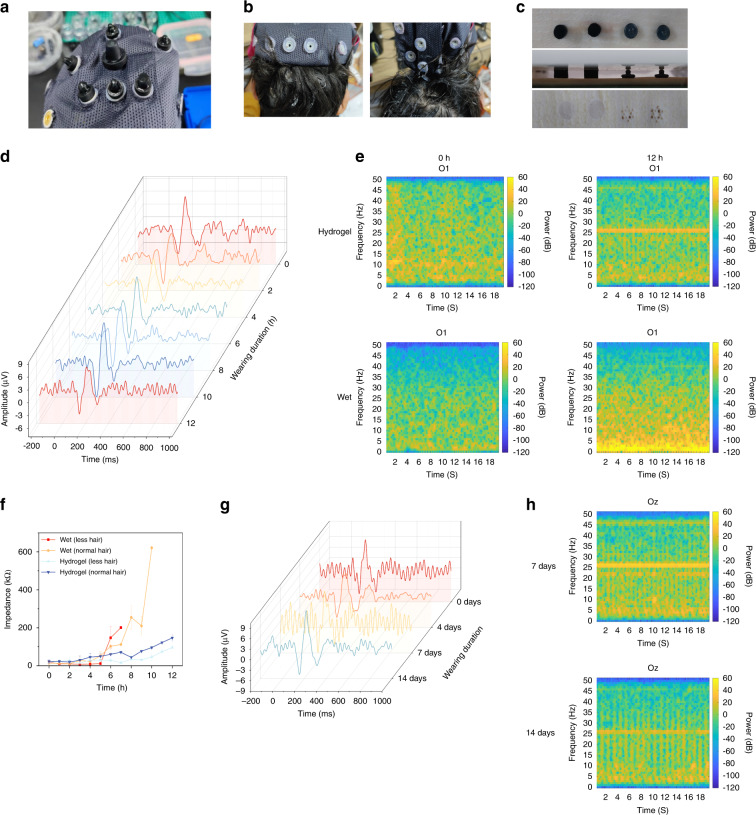
Long-term stable semidry hydrogel electrodes with conductive and substrate-adhesive layers for multiple EEG recordings. **a** Semidry hydrogel electrodes with conductive and substrate-adhesive layers mounted on EEG caps. **b** Pictures of wet electrodes removed after 12 h of continuous wear. **c** The condition of pig skin after the double-layer hydrogel electrodes and dry electrodes were placed for 12 h (under 300 g weight). **d** N170 waves at the O_Z_ for double-layer hydrogel electrodes that were worn for 12 h. **e** PSD of hydrogel and wet electrodes that were worn for 12 h. **f** Real-time impedance of the wet electrodes and hydrogel electrodes detected by the EEG headset with 12 h of continuous recording. **g** N170 waves at the O_Z_ for conductive hydrogel electrodes that were used for 14 days (wore for 2 h a day). **h** PSD of conductive hydrogel electrodes that were used for 14 days (wore for 2 h a day)

The comfortability of the electrode is also an important factor that should be considered for the user. For our double-layer hydrogel electrodes, no discomfort was reported from the subjects tested, and no residues of hair or skin were found on the electrode after long-term usage. The hydrogel electrodes are easily mounted on the EEG cap, featuring an easy-to-go setup (Fig. 7a). On the other hand, wet electrodes require the injection of conductive paste, which causes discomfort and messiness to the subjects’ scalp (Fig. 7b). For solid-pin dry electrodes, discomfort, and pain are reported on the subjects’ heads. In addition, we performed long-term pressure tests using hydrogel and dry electrodes on animal skins. After 12 h of application, hydrogel electrodes did not cause any damage to the skin, whereas solid-pin electrodes inflicted considerable injury to the skin (Fig. 7c).

Whether the hydrogel electrode can be used long-term and repeatedly determines whether it can truly be applied in real-world scenarios. Therefore, we performed a stability test using the ANT neuro EEG cap for continuous EEG acquisition. In a long-term N170 test, we recorded the EEG signal with the conductive hydrogel on a human subject continuously for 12 h (Fig. 7d). The waveforms recorded in this period of time are very similar, with no obvious difference between the signals at hour 0 and hour 12. For the recorded PSD, the hydrogel electrodes showed consistency between 0 and 12 h, whereas the wet electrodes demonstrated a considerable increase in power density after 12 h, particularly at low frequency (Fig. 7e). The real-time impedance is also monitored for both wet and hydrogel electrodes in this period of time. After the hydrogel electrode was worn continuously for 3 h, the skin contact impedance was still comparable to that of the wet electrode. However, after 6 h of continuous wearing, the real-time impedance of wet electrodes increased sharply, and after 8 h, the impedance became infinitely large due to the drying out of the paste (Fig. 7f). In the meantime, the real-time impedance of the hydrogel electrode remains under 150 kΩ for the 12-h period. Additionally, to test if the hydrogel electrode is ready for recycled usage, we performed an N170 test over a period of 14 days with repeated usage of the hydrogel electrode. Even after 14 days of continuous usage, the hydrogel electrodes can still function normally, as indicated by the recorded ERPs. The PSD of the recorded EEG also shows a resemblance after repeated usage (Fig. 7h).

### Limitations

The developed conductive-adhesive double-layer hydrogel electrode shows good robustness, high substrate-bonding strength, low electrode-skin contact impedance, easy-to-go setup, recycled usage, and the ability to acquire EEG in the long term. A comparison of these properties found in hydrogel materials developed by other laboratories is given in Table S1. However, the water retention ability of the conductive hydrogel material here has the potential to be further improved since hydration at the electrode-skin interface is critical to reduce impedance. Additionally, the ability of the hydrogel material to inhibit bacterial growth was not investigated here. Future work should address the bacterial inhibition capability as the hydrogel electrode is subjected to recycled usage on the human scalp.

## Conclusion

In summary, we report a semidry double-layer hydrogel electrode that is convenient to operate, capable of recycled use, and can record high-quality EEG for a prolonged period of time. The conductive hydrogel layer in the electrode is composed of a dual crosslinking network with mobile ions as the conductive medium, whereas the adhesive hydrogel layer can firmly attach to the substrate, eliminating the susceptibility to motion artifacts and preventing the hydrogel from falling off in wearing conditions. The assembled electrode has low skin-contact impedance and can acquire high-fidelity EEG on a scalp with hair. In addition, the hydrogel electrode shows excellent biocompatibility, causing no harm to user skins. It is demonstrated in both N170 and P300 tests that the hydrogel electrode can detect microvolt-level EEG signals and is able to capture ERPs on human subjects, with the recorded signal showing a power spectrum similar to that of wet electrodes. Most importantly, the hydrogel electrodes can detect EPRs continuously for up to 12 h, whereas the wet electrode loses function after 4 h. Overall, the developed double-layer hydrogel electrode has the potential to extend the application of noninvasive BCIs to numerous daily scenarios owing to its convenience of operation and the ability to collect high-quality EEGs in the long term.

## Supplementary information

supporting information
