# Supplementary material for: Hydrogel electrodes with conductive and substrate-adhesive layers for noninvasive long-term EEG acquisition
Source: Microsyst Nanoeng. 2023 Jun 12;9:79. doi: 10.1038/s41378-023-00524-0 (PMC10258200; doi:10.1038/s41378-023-00524-0)
Supplement: Supplementary file 1 — supporting information [file 41378_2023_524_MOESM1_ESM.docx]

Table S1. Summary of performance of some hydrogels with both mechanical robustness and adhesion.

| Conductivity | Conducting manner | Contacting impedance/ resistance | Water retention | Adhesiveness  (Against glass) | Biocompatibility | Reference |
| --- | --- | --- | --- | --- | --- | --- |
| 0.076 ~ 0.099 S/m | Free ions from clay | 105 kΩ | No test | 16.8 kpa | No test | 1 |
| 0.16 S/m  (0.01-10^5^ Hz) | Mobile ions | No test | No test | 11.1 kpa | No skin irritation and inflammation | 2 |
| No test | Polymer backbone | ~0.2 S/m in PBS, ~0.4 S/m in deionized water | Dried in room condition.  Re-hydrolyze with water | No test | No test | 3 |
| 100~1200 kΩ  （1~50 Hz） | Silver nanowires | 2.98~155.5 Ω /sq | No test | No test | No skin inflammation | 4 |
| ≤6 kΩ  (1~50Hz) | Mobile ions | ~8.18  (EEG 0.5~50Hz) | No test | No test | No cytotoxicity  No inflammatory responses | 5 |
| No test | Silver nanowires | ≤10 kΩ  (EEG 0.5~50Hz) | No test | No test | No test | 6 |
| ≤3.8 kΩ  (1~50Hz) | Mobile ions | ~5 kΩ  (EEG 0.5~50 Hz) | Weigh stable after 7 days. Retains > 50% water content | 63 kpa | No cytotoxicity  No skin irritation and inflammation (Rabbit) | This work |

**References:**

1. Wang, L. *et al.* Tough, Adhesive, Self-Healable, and Transparent Ionically Conductive Zwitterionic Nanocomposite Hydrogels as Skin Strain Sensors. *ACS Appl. Mater. Interfaces* **11**, 3506–3515 (2019).

2. Zhao, H. *et al.* Ultrafast Fabrication of Lignin-Encapsulated Silica Nanoparticles Reinforced Conductive Hydrogels with High Elasticity and Self-Adhesion for Strain Sensors. *Chem. Mater.* **34**, 5258–5272 (2022).

3. Lu, B. *et al.* Pure PEDOT:PSS hydrogels. *Nat. Commun.* **10**, 1043 (2019).

4. Yang, X. *et al.* Ultrathin, Stretchable, and Breathable Epidermal Electronics Based on a Facile Bubble Blowing Method. *Adv. Electron. Mater.* **6**, 2000306 (2020).

5. Ju, Y. Leaf-inspired homeostatic cellulose biosensors. *Sci. Adv.* (2021).

6. Chen, C. *et al.* Novel Flexible Material-Based Unobtrusive and Wearable Body Sensor Networks for Vital Sign Monitoring. *IEEE Sens. J.* **19**, 8502–8513 (2019).
